# Magnitude, Pattern, and Associated Factors of Thyroid Dysfunction in Diabetic Patients at Debre Markos Referral Hospital in North-West Ethiopia: A Cross-Sectional Hospital-based Study

**DOI:** 10.4314/ejhs.v35i2.9

**Published:** 2025-03

**Authors:** Melkamu Tilahun

**Affiliations:** 1 Department of Biomedical Sciences, College of Medicine and Health Sciences, Debre Markos University, Debre Markos, Ethiopia

**Keywords:** Diabetes mellitus, thyroid dysfunction, diabetic complications

## Abstract

**Background:**

Diabetes mellitus and thyroid disease are the two most prevalent endocrine disorders recognized in clinical practice in the twenty-first century. Diabetic patients with undiagnosed thyroid disorders are twice as likely to suffer from diabetic complications. The relationship between thyroid dysfunction and its risk factors in diabetic patients in Ethiopia is still being explored. We aimed to assess the magnitude, patterns, and associated factors of thyroid dysfunction in diabetic patients at Debre Markos Referral Hospital.

**Methods:**

The study involved 426 participants, selected using a systematic sampling procedure. Pregnant women, individuals with neck radiation exposure, and those who had undergone thyroid surgery were excluded. Measurements including weight, height, blood pressure (BP), fasting blood glucose (FBG), triiodothyronine (T3), thyroxine (T4), thyroid stimulating hormone(TSH), total cholesterol, and triglyceride levels were taken. Data were entered into EpiData 3.1 and exported to SPSS for analysis. Variables with a p-value of <0.25 in bivariable logistic regression were included in multivariate logistic regression. Variables with a p-value <0.05 in multivariate logistic regression were considered statistically significant.

**Results:**

Thyroid dysfunction was found in 102 (23.94%) of the respondents, with 29 (6.8%) diagnosed with hyperthyroidism and 73 (17.1%) with hypothyroidism. The majority of those with thyroid dysfunction (11.50%) had clinical hypothyroidism. Glycemic control, illness duration, blood cholesterol, and low-density lipoprotein (LDL) were significantly associated with thyroid dysfunction.

**Conclusion:**

Thyroid problems were present in 25% of diabetic patients. Thyroid dysfunction was associated with poor glycemic control, long-term illness, high blood cholesterol, and low-density lipoprotein levels.

## Introduction

Diabetes is one of the most significant global health risks of the twenty-first century. With over 452 million cases reported in 2017 and an estimated 694 million cases by 2045, diabetes continues to grow as a global health issue (1). Diabetes affects nearly every part of the body, causing complications such as blindness, metabolic disorders, cardiovascular disease, stroke, kidney failure, and nerve damage (2). The cost of diabetes care amounted to over 9.5 billion USD in 2019, accounting for 1% of global health expenditure (3).

Thyroid disease and diabetes mellitus are the most common endocrine disorders encountered in clinical practice. Thyroid hormones are involved in regulating carbohydrate metabolism and pancreatic function; however, diabetes can significantly impact thyroid function testing (4,5).

Diabetes can cause fluctuations in thyroid hormone levels. Insulin mimics the effects of thyroid hormone in many biological tissues while acting in opposition in others (6). Diabetes can impair thyroid function by altering thyroid-stimulating hormone (TRH), the conversion of T4 to T3 in peripheral tissues, and thyroid gland function. Both excess and deficiency of insulin can affect thyroid hormone production and function. Conversely, thyroid dysfunction can contribute to insulin resistance. Understanding the link between diabetes and thyroid disease can aid clinicians in screening for and treating both conditions simultaneously (7–9).

Thyroid disease is a global health issue, accounting for 30-40% of all endocrine disorders (10). Thyroid dysfunction is common in the general population, with prevalence rates ranging from 6.6% to 13.4%. However, it is more common in diabetic patients, with rates ranging from 10% to 24% (11). Thyroid dysfunction is more prevalent in type 1 diabetes than in type 2 diabetes (12). Undiagnosed thyroid dysfunction can have adverse effects on diabetes and its related complications (13).

The diagnosis of subclinical thyroid dysfunction is often delayed due to subtle clinical symptoms (14). Although Africa accounts for 25% of the global disease burden, the overall incidence of thyroid disorders in diabetics is not well understood, particularly in Ethiopia. This study aimed to evaluate the magnitude, patterns, and factors associated with thyroid abnormalities in diabetic patients at Debre Markos Referral Hospital in North-West Ethiopia.

## Methods

**Study design and period**: An institutional-based cross-sectional follow-up study was conducted on diabetic patients. A total of 426 diabetic patients were recruited from the diabetes clinic at Debre Markos Referral Hospital. Data were collected between April and May of 2021.

**Study area**: The study was conducted at Debre Markos Comprehensive Specialized Hospital (DMCSH), located in Debre Markos town, with coordinates of 10°20′N, 37°43′E, and an elevation of 2,446 meters. Debre Markos is situated in the northwestern part of Ethiopia, 265 km from Bahir Dar and 300 km from Addis Ababa. Debre Markos Comprehensive Specialized Hospital is the only referral hospital in the East Gojjam Zone, serving approximately 3.5 million people. Its referral center operates seven days a week.

**Eligibility criteria**: All diabetic patients who visited the Diabetes Clinic at Debre Markos Referral Hospital for a check-up were included in the study. However, diabetic patients who had undergone thyroid surgery, experienced neck trauma, had a history of previous neck radiation exposure, were taking medications (such as amiodarone, lithium, interferon alpha, iodides, beta-blockers, carbimazole, or propylthiouracil), were pregnant, or had severe illness were excluded.

**Sample size determination and sampling procedures**: The sample size was determined using the single population proportion formula, considering a 50% prevalence of thyroid dysfunction, a 5% margin of error, a 95% confidence level, and a 10% non-response rate. The final sample size calculation yielded 426 participants.

The patient register was used as the sample frame. The first participant was randomly selected from the first five patients (2321/426 = 5). Subsequent participants were chosen at five intervals based on the order of their medical records until the required sample size (426) was met.

### Operational definitions

**Primary Hypothyroidism**: High TSH (>5 µU/ml) and low free T3 (<4.0 pmol/L) and free T4 (<10.6 pmol/L) (15-18).

**Subclinical Hypothyroidism**: High TSH (>5 µU/ml) but normal free T3 (4.0–8.3 pmol/L) and free T4 (10.6–19.4 pmol/L) (15-18).

**Primary Hyperthyroidism**: Low TSH (<0.25 µU/ml) and high free T3 (>8.3 pmol/L) and free T4 (>19.4 pmol/L) (15-18).

**Subclinical Hyperthyroidism**: Low TSH (<0.25 µU/ml) but normal free T3 (4.0–8.3 pmol/L) and free T4 (10.6–19.4 pmol/L) (15-18).

**Secondary Hypothyroidism**: TSH <0.25 µU/ml or a normal TSH (0.25–5 µU/ml) with low free T3 (<4.0 pmol/L) and free T4 (<10.6 pmol/L) (15-18).

**Secondary Hyperthyroidism**: High TSH (>5 µU/ml) with low free T3 (<8.3 pmol/L) and free T4 (10.6–19.4 pmol/L) (15-18).

**Poor Glycemic Control**: An average fasting blood sugar level of >130 mg/dl over the past six months;

**Good Glycemic Control**: An average fasting blood sugar level of <130 mg/dl over the past six months (19).

**Data collection procedure and measurement of study variables**: Three clinical nurses and three laboratory technologists collected the data. Each participant underwent a face-to-face interview, anthropometric measurements, thyroid function tests, and lipid profile tests. Data were also extracted from medical records using a pre-tested structured checklist.

**Diabetes mellitus**: Diabetes was diagnosed according to American Diabetes Association (ADA) criteria, with type I and type II diabetes distinguished based on the age of onset and reliance on insulin therapy (20).

**Blood pressure**: Hypertension was defined as a systolic blood pressure ≥140 mm Hg and/or diastolic blood pressure ≥90 mm Hg in two consecutive measurements taken four hours apart, along with the use of antihypertensive medication (21).

**Anthropometric data**: Height was measured on a standardized measuring board (stadiometer), and weight was measured using a computerized scale after participants removed their shoes and heavy clothing. BMI was calculated as weight (kg) divided by height (m^2^).

**Biochemical tests**: A 5 ml morning venous blood sample was collected for TSH, free T3, free T4, and lipid profiles. Blood glucose levels were determined from an average of six follow-up fasting blood sugar tests (19). Serum samples were centrifuged and analyzed for thyroid hormone levels using an enzyme-linked immunoassay method (VIDAS, Biomeriux SA, France) and for lipid profiles using an enzymatic technique (ABX Pentra 400, France) (22).

**Data quality management**: The tool was reviewed by senior experts, and a pre-test was conducted at Felege Hiwot Specialization Hospital. The questionnaire was revised based on the pre-test results. To ensure data quality, regular supervision was provided, and relevant materials were developed and implemented. The questionnaire was translated from English to Amharic and back to English for accuracy. Data collectors received two days of training before data collection began. Data quality was ensured through consistent checks for completeness and consistency during data collection, management, storage, cleaning, and analysis. The principal investigator verified the quality of the data prior to analysis.

**Data processing and analysis**: The data were examined, classified, and encoded before being entered into EpiData version 3.1. Statistical analysis was performed using SPSS version 25. Descriptive statistics were used to determine the frequency, mean, percentage, and standard deviations. Cross-tabulation was used to analyze the relationship between dependent and independent variables. Binary logistic regression was used to assess associations between independent variables and thyroid dysfunction, with a 95% confidence interval. Variables with p-values <0.25 in bivariate logistic regression were included in multivariate logistic regression, and those with p-values <0.05 were considered statistically significant. Results were presented in text, tables, charts, and graphs.

**Ethics approval and consent to participate**: This study was conducted in accordance with the updated Declaration of Helsinki guidelines for biomedical research involving human participants (38). Ethical approval was granted by the Debre Markos University Institutional Ethical Review Board. Additionally, Debre Markos Referral Hospital provided a letter of support for the study. Data collection commenced only after obtaining informed consent from the participants. Study participants were informed of their right to decline participation, ask questions, and withdraw from the study at any time. The confidentiality and privacy of the participants were ensured throughout the study. Following the diagnosis of macula edema, participants were referred to the eye clinic for further treatment.

## Results

**Socio-demographic characteristics of the respondents**: A total of 426 diabetic individuals participated in the study, with a 100% response rate. The average age was 43.20 years (±16.40). Two-thirds of the respondents (63.84%) were male. More than half (71.95%) of the participants were aged over 40. Two-thirds of the respondents were married (61.74%), and the majority resided in urban areas (62.14%). Nearly half of the respondents (42.98%) had a bachelor's degree or higher, while one-third (33.57%) worked for the government or a non-governmental organization. About one-third of the participants (31.22%) had a family history of diabetes mellitus. Additionally, 5.40% of patients had macular edema (Table 1).

**Table 1:** Socio-demographic features of diabetic patients on follow-up at Debre Markos Referral Hospital, North West Ethiopia in 2021

Variables (N=426)	Thyroid dysfunction		Total number (%)
	Yes (number (%)	No (Number (%)	
Sex			
Female	41(9.62)	97(22.77)	138(32.40)
Male	61(14.32)	227(53.29)	288(67.61)
Age			
≤40 years	37(8.69)	159(37.32)	196(46.01)
>40 years	65(15.26)	165(38.73)	230(53.99)
Religions			
Orthodox	94(22.07)	300(70.42)	394(92.49)
Muslim	8(1.89)	24(5.63)	32(7.51)
Place of Residency			
Rural	16(3.76)	86(20.19)	102(23.94)
Urban	86(20.19)	238(55.87)	324(76.06)
Marital status			
Single	5(1.17)	64(15.02)	69(16.20)
Married	52(12.21)	227(53.29)	279(65.49)
Divorced	11(2.59)	12(2.82)	23(5.39)
Widowed	34(7.99)	21(4.93)	55(12.91)
Educational status			
No formal education	12(2.82)	66(15.50)	78(18.21)
Primary-level	11(2.59)	89(20.90)	100(23.51)
Secondary -level	13(3.05)	43(10.10)	56(13.25)
College education	66(15.50)	126(29.58)	192(45.03)
Occupation			
Merchant	39(9.15)	114(26.76)	153(35.92)
Employee	36(8.45)	113(26.53)	149(34.97)
Farmer	19(4.46)	61(14.32)	80(18.78)
Student	8(1.88)	36(8.45)	44(10.33)
Family history of TD			
No	83(19.48)	286(67.14)	369(86.61)
Yes	19 (4.46)	38(8.92)	57 (13.38)

**Behavioral, clinical, and diabetic care characteristics of the respondents**: The prevalence of cigarette smoking among respondents was 0.7%, and 17.37% reported alcohol consumption. Nine out of ten respondents (88.73%) did not engage in regular physical activity, although almost all (92.49%) consumed adequate iodine. The average body mass index (BMI) was 25.79 ± 2.9 kg/m^2^. Approximately three-quarters (71.3%) of the participants were of normal weight, while one-quarter (22.6%) were either overweight or obese. A quarter of the respondents (23%) reported a history of hypertension. The mean fasting blood glucose level was 136.06 ± 28.86 mg/dl, and 50.70% of respondents had adequate glycemic control. Nearly half of the participants (53.52%) used oral antiglycemic medication, while 41.55% used insulin. Three-quarters of the patients (74.5%) adhered to their prescribed therapies. Additionally, six out of ten patients (61.03%) reported attending a hospital once a month for diabetic follow-up (see Table 2 for further details).

**Table 2:** Behavioural, clinical, and diabetic care characteristics of respondents at Debre Markos Referral Hospital, North West Ethiopia, in 2021

Variables (N = 426)	Thyroid dysfunction		Total Number (%)
	Yes (Number (%)	No (Number (%)	
Alcohol consumption			
Never drink after Dx	78 (18.31)	274 (64.32)	352 (82.63)
Drinker	24 (5.63)	50 (11.74)	74 (17.37)
Physical exercise			
No	90(21.13)	288(67.61)	378(88.73)
Yes	12(2.82)	36 (8.45)	48 (11.27)
Iodine intake			
No	94(22.07)	300 (70.42)	394(92.49)
Yes	8(1.89)	24(5.63)	32(7.51)
Duration of illness			
≤10 years	30 (7.04)	239 (56.10)	269 (63.15)
>10 years	72 (19.90)	85(19.95)	157 (36.85)
Glycemic control level			
Good control	19 (4.46)	197(46.24)	216 (50.70)
Poor control	83 (19.48)	127(29.81)	210 (49.26)
Hypertension			
No	43 (10.09)	285(66.90)	328 (77.00)
Yes	59 (13.85)	38(9.15)	98 (23.00)
Treatment Modality			
Oral agent alone	60(14.08)	168(39.44)	228(53.52)
Insulin alone	33(7.75)	144(33.08)	177(41.55)
Both	9(2.11)	1(2.82)	21(4.93)
Cardiac illness			
No	96 (22.54)	330 (77.46)	402 (94.37)
Yes	6 (1.41)	8 (1.88)	14 (3.29)
Chronic kidney disease			
No	91(21.36)	335(78.64)	409(96.00)
Yes	11(2.58)	6(1.41)	17(4.00)
Treatment adherence			
Good	58(13.62)	275(64.55)	333(76.17)
Poor	44(10.33)	49(11.50)	93(21.83)
Follow up frequency			
Every month	63(14.79)	197(46.24)	260(61.03)
Every two months	39(9.15)	127(29.81)	166(38.97)

**Biochemical parameters of the respondents**: Approximately 46.24% of the participants had normal high-density lipoprotein (HDL) levels, while 49.06% had abnormal low-density lipoprotein (LDL) values. Thyroid function tests were normal for three-quarters of the participants (76.1%), but one-quarter (23.9%) exhibited various forms of thyroid dysfunction Tables 3 and 4).

**Table 3:** Lipid profile characteristics of respondents at Debre Markos Referral Hospital in North West Ethiopia, 2021

Variables (N = 426)	Thyroid dysfunction		Total Number (%)
	Yes (Number (%)	No (Number (%)	
Total blood cholesterol			
Normal value	18 (4.22)	192(45.07)	210(49.30)
Abnormal value	82(19.24)	134 (31.45)	216 (50.70)
High-density lipoprotein			
Normal value	11 (2.58)	186(43.66)	197 (46.24)
Abnormal value	91(21.36)	138(32.39)	229 (53.76)
Low-density lipoprotein			
Normal value	24 (5.63)	193(45.31)	217(50.93)
Abnormal value	78(18.31)	131(30.75)	209 (49.06)
Triglycerides			
Normal value	43 (10.09)	285(66.90)	328 (77.00)
Abnormal value	59 (13.85)	38(9.15)	98 (23.00)

**Table 4:** Thyroid function findings for respondents at Debre Markos Referral Hospital, North West Ethiopia, 2021

Thyroid function (N= 426)	T1DM (n = 187)	T2DM (n = 239)	Total
Euthyroidism	151(35.45%)	173(40.14%)	324 (76.1%)
Sub clincal-Hypothyrodesm	6(1.41%)	11(2.58%)	17 (4%)
Clinical-Hypothyroidism	18(4.23%)	31 (7.28%)	49 (11.50%)
Overt- Hypothyroidism	2(0.47%)	5(1.17%)	7(1.64%)
Sub clinical- Hyperthyroidism	3(0.7%)	7(1.64%)	10(2.35%)
Clinical- Hyperthyroidism	5(1.17%)	14(3.28%)	19(4.46%)

**Figure 1:**
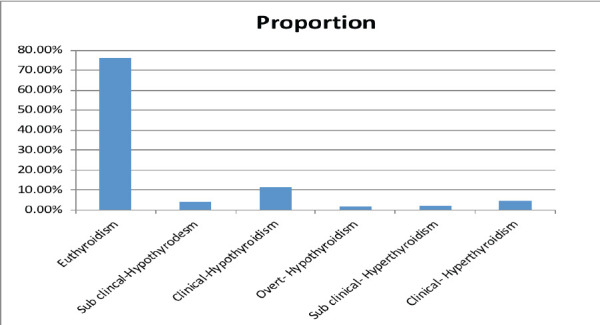
Thyroid function test results for respondents at Debre Markos Referral Hospital, North West Ethiopia, 2021

**Prevalence of thyroid dysfunction**: Thyroid dysfunction was identified in 102 (23.94%) individuals. Of the participants, 324 (76.1%) were euthyroid, 29 (6.8%) had hyperthyroidism, and 73 (17.1%) had hypothyroidism. Among those with hypothyroidism, 11.5% had clinical hypothyroidism, 4.0% had subclinical hypothyroidism, and 1.64% had overt hypothyroidism (Figures 1).

**Associated factors for thyroid dysfunction among diabetic patients**: Bivariable logistic regression results with p-values <0.25 were included in the multivariable logistic regression analysis. Glycemic control, disease duration, total blood cholesterol, and LDL were found to be statistically significant risk factors for thyroid dysfunction. Diabetic individuals with poor glycemic control had a threefold higher risk of developing thyroid dysfunction (AOR (95% CI): 3.6 (1.70, 11.10)) compared to those with good glycemic control. Patients with a disease duration of more than ten years were four times more likely (AOR (95% CI): 4.2 (2.58, 7.60)) to develop thyroid dysfunction compared to those with less than ten years of illness. Those with abnormal total blood cholesterol were twice as likely (AOR (95% CI): 2.5 (1.07, 5.11)) to have thyroid dysfunction than those with normal cholesterol levels. Additionally, patients with abnormal LDL levels had a threefold increased risk (AOR (95% CI): 3.01 (2.34, 9.50)) of thyroid dysfunction compared to those with normal LDL levels (Table 5).

**Table 5:** Multivariate analysis of clinical, diabetic care, and serum biochemical characteristics among respondents at Debre Markos Referral Hospital, North West Ethiopia in 2021

Variables (N= 426)	TD		COR with 95% CI	AOR with 95% CI	P-value

	Yes	No			
Glycemic control level					
Good	19	197	1	1	
Poor	83	127	0.15 (0.13,0.56)	3.6(1.70,11.10)	.003*
Hypertension					
No	43	285	1	1	
Yes	59	38	0.09 (0.04,0.91)	2.80 (0.29,6.07)	.067*
Treatment modality					
Oral agent alone	60	168	1	1	
Insulin alone	33	144	0.36 (0.19, 0.69)	1.23(.48,3.18)	.666
Both	9	1	1.5 (0.13,16.97)	.81(.03,24.31)	.903
Duration of DM					
≤10 years	19	197	1	1	
> 10 years	83	127	0.14(0.11,0.82)	4.2 (2.58,7.60)	.002*
Total blood cholesterol					
Normal value	18	192	1	1	
Abnormal value	82	134	0.70 (0.32,0.84)	2.5 (1.07,5.11)	.032*
Low-density lipoprotein					
Normal value	24	193	1		
Abnormal value	78	131	0.21 (0.12.,0.68)	3.01(2.34,9.50)	.001

(*) Statistically significant at p- value > 0.05

## Discussion

Thyroid dysfunction was identified in approximately one-quarter of diabetic patients (23.94%). This finding aligns with studies from India (31.2%) (23) and Saudi Arabia (28.5%) (24), but is higher than those from Greece (12.3%) (25), Jordan (12.5%) (26), Pedro Ernesto University Hospital (14.7%) (27), and Iraq (16%) (28). However, the prevalence observed in this study is lower than that reported in Nepal (36%) (3) and Spain (34.8%) (22). These differences may be attributed to factors such as genetics, methodology, study setting, comorbidities, diagnostic techniques, quality of care, and health-seeking behavior of the participants.

Hypothyroidism was the most prevalent form of thyroid dysfunction (17.1%), while hyperthyroidism accounted for 6.8%. Among the hypothyroid participants, clinical hypothyroidism comprised 11.5%, subclinical hypothyroidism 3.9%, and overt hypothyroidism 1.6%. These findings are consistent with a study conducted in Saudi Arabia, which reported 15.3%, 9.5%, and 0.5% for clinical, subclinical, and overt hypothyroidism, respectively. Similarly, the prevalence of hyperthyroidism in Saudi Arabia was 5.9%, which is in line with our findings (24). These results are higher than those found in studies from Iraq (7%) (28) and Spain (9.5%) (22). These discrepancies could be due to differences in the quality of care provided to diabetic patients, risk factors, or diagnostic methods employed.

The multivariable logistic regression analysis identified glycemic control, disease duration, total blood cholesterol, and LDL levels as statistically significant predictors of thyroid dysfunction. Our study found that individuals with poor glycemic control had a threefold higher risk of thyroid dysfunction (AOR (95% CI): 3.6 (1.70, 11.10)) compared to those with good control. This is consistent with research conducted at the University of Nigeria Teaching Hospital, where hyperglycemia was associated with dysregulation of the hypothalamus-pituitary-thyroid axis, which may lead to low T3 levels in diabetic patients. This could be due to hyperglycemia-induced inhibition of peripheral T4 to T3 deiodination (25,29).

We also confirmed the relationship between the duration of diabetes and thyroid dysfunction. Individuals with a disease duration of more than ten years had a fourfold increased likelihood of developing thyroid dysfunction (AOR (95% CI): 4.2 (2.58, 7.60)) compared to those with a shorter duration. This finding aligns with studies from Iraq (28), Jordan (30), and Spain (22), suggesting that prolonged hyperglycemia can impair T4 to T3 deiodination, leading to thyroid dysfunction (31).

Our study also found a significant association between dysregulation of total blood cholesterol and thyroid dysfunction. This finding is consistent with research from Chubb (32), Zhang (33), Turkey (34), Brazil (35), and Korea (36). Thyroid hormones play a role in liver cholesterol metabolism; when thyroid hormone levels are low, cholesterol metabolism is slowed, leading to higher cholesterol levels in the blood (37). Additionally, the significant correlation between LDL levels and thyroid dysfunction in our study (AOR (95% CI): 3.01 (2.34, 9.50)) further supports this idea, as thyroid hormones may facilitate hepatic cholesterol metabolism. Low thyroid hormone levels result in slower cholesterol processing, which can increase cholesterol levels in the bloodstream (37).

In conclusion, these findings suggest that thyroid dysfunction is highly prevalent among diabetic individuals. Glycemic control, illness duration, blood cholesterol, and low-density lipoprotein (LDL) were significantly associated with thyroid dysfunction.
